# Initiation of Chromosomal Replication in Predatory Bacterium *Bdellovibrio bacteriovorus*

**DOI:** 10.3389/fmicb.2016.01898

**Published:** 2016-11-28

**Authors:** Łukasz Makowski, Rafał Donczew, Christoph Weigel, Anna Zawilak-Pawlik, Jolanta Zakrzewska-Czerwińska

**Affiliations:** ^1^Department of Microbiology, Hirszfeld Institute of Immunology and Experimental Therapy – Polish Academy of SciencesWrocław, Poland; ^2^HTW, Department of Life Science EngineeringBerlin, Germany; ^3^Department of Molecular Microbiology, Faculty of Biotechnology, University of WrocławWrocław, Poland

**Keywords:** *oriC*, DnaA, initiation of chromosome replication, *Bdellovibrio bacteriovorus*, *Escherichia coli*, *Pseudomonas putida*

## Abstract

*Bdellovibrio bacteriovorus* is a small Gram-negative predatory bacterium that attacks other Gram-negative bacteria, including many animal, human, and plant pathogens. This bacterium exhibits a peculiar biphasic life cycle during which two different types of cells are produced: non-replicating highly motile cells (the free-living phase) and replicating cells (the intracellular-growth phase). The process of chromosomal replication in *B. bacteriovorus* must therefore be temporally and spatially regulated to ensure that it is coordinated with cell differentiation and cell cycle progression. Recently, *B. bacteriovorus* has received considerable research interest due to its intriguing life cycle and great potential as a prospective antimicrobial agent. Although, we know that chromosomal replication in bacteria is mainly regulated at the initiation step, no data exists about this process in *B. bacteriovorus*. We report the first characterization of key elements of initiation of chromosomal replication – DnaA protein and *oriC* region from the predatory bacterium, *B. bacteriovorus*. *In vitro* studies using different approaches demonstrate that the *B. bacteriovorus oriC* (Bd*oriC*) is specifically bound and unwound by the DnaA protein. Sequence comparison of the DnaA-binding sites enabled us to propose a consensus sequence for the *B. bacteriovorus* DnaA box [5′-NN(A/T)TCCACA-3′]. Surprisingly, *in vitro* analysis revealed that Bd*oriC* is also bound and unwound by the host DnaA proteins (relatively distantly related from *B. bacteriovorus*). We compared the architecture of the DnaA–*oriC* complexes (orisomes) in homologous (*oriC* and DnaA from *B. bacteriovorus*) and heterologous (Bd*oriC* and DnaA from prey, *Escherichia coli* or *Pseudomonas aeruginosa*) systems. This work provides important new entry points toward improving our understanding of the initiation of chromosomal replication in this predatory bacterium.

## Introduction

*Bdellovibrio* are small intriguing Gram-negative predatory bacteria that enter and kill other Gram-negative bacteria, including many pathogens, such as *Campylobacter*, *Helicobacter* (Markelova, 2010), *Escherichia* (Varon and Shilo, 1968), *Pseudomonas, Salmonella* (Iebba et al., 2014), *Fusobacterium nucleatum*, and *Aggregatibacter actinomycetemcomitans* (a member of oral microbial communities) (Loozen et al., 2015). The widespread species of this genus is *Bdellovibrio* *bacteriovorus*, which inhabits a wide range of environments, including fresh water, sewage, soil, and even mammalian intestines (Rendulic et al., 2004). *B.* *bacteriovorus* is a small bacterium (0.2–0.5 μm wide and 0.5–2.5 μm long) that possesses a relatively large 3.85-Mb genome that encodes many predation-associated proteins, such as proteases, peptidases, and other hydrolytic enzymes.

*Bdellovibrio bacteriovorus* exhibits a biphasic lifecycle consisting of a free-living non-replicative attack phase and an intracellular growth phase (Sockett, 2009). In the free-living phase, this highly motile bacterium searches for its prey; after attaching to the prey’s outer membrane, it passes through the peptidoglycan layer into the periplasm and begins its intracellular growth phase (Lambert et al., 2008). Inside the periplasm, *B. bacteriovorus* degrades the host’s macromolecules using different types of hydrolytic enzymes, allowing it to grow and replicate its chromosome (Rendulic et al., 2004). This chromosomal replication is not followed by cell division, but instead leads to the formation of a multinucleoid elongated filamentous. When the resources of the host cell are exhausted, the elongated filament synchronously septates to form usually three to six *B. bacteriovorus* progeny cells (Fenton et al., 2010). These progeny cells become motile, and then are released into the environment through lysis of the host cell. Interestingly, *B. bacteriovorus* can also enter (albeit rarely and only in the presence of abundant amino acids and cofactors) into a replicative host-independent phase (Seidler and Starr, 1969). *B. bacteriovorus* has received considerable recent research interest, owing to its intriguing life cycle and its great potential to be applied as an antimicrobial agent in industry, agriculture, and/or medicine. To fully utilize *B. bacteriovorus* in any of these roles, however, we must better understand the cell biology of this pathogen at the molecular level.

Chromosomal replication, which is a key event in the bacterial life cycle, is mainly controlled at the initiation step (Zakrzewska-Czerwińska et al., 2007). In *B. bacteriovorus*, as in other bacteria, the initiation of chromosomal replication is strictly regulated and adjusted with respect to its cell cycle. Replication must be initiated after *B. bacteriovorus* enters the prey, and it must cease before bdelloplast septation to ensure that each cell receives a single copy of the chromosome. However, even the key elements of replication initiation have not yet been identified for *B. bacteriovorus*.

In bacteria, replication begins at a single chromosome site called the origin of replication (*oriC*). The process is initiated through the cooperative binding of the initiator protein, DnaA, to specific 9-mer sequences (called DnaA boxes) within the *oriC* region. This causes the DNA strands to separate at the AT-rich DNA unwinding element (DUE), allowing the entry of helicase and, later, other enzymes required for DNA synthesis (e.g., primase and DNA Pol III). Bacterial origins, which may be a continuous unit or divided in two parts (bipartite *oriC*), range in length from -200 to 1000 bp or longer (when they are split). They can differ in various characteristics, including the numbers, orientations, and sequences of their DnaA boxes, and the localizations and sequences of the AT-rich regions and other motifs, including those recognized by regulatory proteins. The various modules (e.g., DnaA boxes, the DUE, etc.) constitute the central management system responsible for forming the functional initiation complex (orisome) and/or regulating the assembly of this complex (Leonard and Grimwade, 2015; Wolański et al., 2015).

Here, we report the first characterization of DnaA and *oriC* from the predatory bacterium, *B. bacteriovorus.* We demonstrate that the *B*. *bacteriovorus oriC* (Bd*oriC*) is specifically bound and unwound not only by its own DnaA, but surprisingly also by the host’s DnaA proteins.

## Materials and Methods

### Bacterial Strains and Growth Conditions

The wild-type *B. bacteriovorus* strain HD100 (Rendulic et al., 2004) and the axenic *B. bacteriovorus* strain HI (Roschanski et al., 2011) were used in this study. *B. bacteriovorus* HD100 was grown at 30°C by predation on *Escherichia coli* S-17 in HEPES buffer (25 mM HEPES, 2 mM CaCl_2_, 3 mM MgCl_2_, pH 7.8) and 200 rpm, or on double-layer plates [bottom layer – YPSC medium (0.1% Yeast Extract, 0.1% Pepton, 0.05% Sodium Acetate, 0.025% Magnesium Sulfate; pH 7.6] with 1% agar, top layer – YPSC with 0.6% agar and supplemented with *E. coli* S-17 liquid culture, both layers were supplemented with 0.025% CaCl_2_ after autoclaving). *E. coli* was grown in LB medium (liquid or agar) at 37°C. *B. bacteriovorus* HI was grown in PYE medium (1% Bacto Peptone, 0.3% yeast extract, 2 mM CaCl_2_, 3 mM MgCl_2_, pH 7.6) at 30°C and 200 rpm.

### *In silico* Origin Prediction

The *oriC*-type replication origins in the genomes of *B. bacteriovorus* HD100 [GenBank entry BX842601.2], *B. bacteriovorus* str. Tiberius [GenBank entry CP002930.1], *Bdellovibrio exovorus* JSS [GenBank entry CP003537.1], and *Halobacteriovorax marinus* SJ [GenBank entry FQ312005.1] were predicted using the following stepwise procedure: (1) The annotation of the *dnaA* gene in the genome was validated by TBLASTN (version 2.2.30) (Shiryev et al., 2007) using the DnaA sequence of *E. coli* K-12 MG1655 [GenBank entry AAC76725.1] as a query. (2) The approximate genomic location of *oriC* was roughly determined based on the inflection point (minimum) of the genome’s cumulative GC-skew, which was obtained from the Comparative Genometrics website (Roten et al., 2002) or the GenSkew webserver^1^ with the following parameter settings: Nucleotide1: G, Nucleotide2: C, Windowsize: 500, Stepsize: 100. (3) WebSIDD (Bi and Benham, 2004)^2^ was used under default settings (37°C, 0.1 M salt, circular DNA, copolymeric) and negative superhelicity values in the range of σ = -0.04 (low) to σ = -0.06 (high) in increments of 0.005, in order to identify putative DUE(s) (Kowalski and Eddy, 1989) in intergenic regions near (±10 kb) of the GC-skew inflection point (minimum). (4) DnaA boxes were assigned manually using the *E. coli* consensus, 5′-TTWTNCACA (Schaper and Messer, 1995), and allowing for three mismatches. (5) A prediction was considered significant if a DnaA box could be assigned to a position of approximately two helical turns distant from the border of a strong DUE. Prediction output data were obtained as raw text files and further processed with Microsoft Excel v97SR-1 and Corel Draw v.11.

### DnaA Purification

The *B. bacteriovorus dnaA* gene was PCR amplified from chromosomal DNA with primers P-1 and P-2 (Supplementary Table S1), cut with BamHI and XhoI and then cloned into the pET28a(+) expression vector linearized with the same restriction enzymes. The 6HisBdDnaA protein was produced in *E. coli* BL21 containing pET28a(+)*BddnaA*. When the culture reached an OD_600_ = 1.9, fusion protein synthesis was induced by addition of 1 mM IPTG, after which cells were incubated for 3 h at 37°C. The bacteria were harvested by centrifugation (10 min, 5000 *g*, 4°C) and the bacterial pellets were stored at -20°C. The purification of 6HisBdDnaA was performed as described previously (Zawilak-Pawlik et al., 2006), except that LG_100_ buffer (45 mM HEPES/KOH, pH 7.6, 100 mM potassium glutamate, 10 mM magnesium acetate, 1 mM DTT and 20% sucrose) was used in place of the LG_200_ buffer.

### Electrophoretic Mobility Shift Assay (EMSA)

The interactions of the DnaA protein with DNA were analyzed as previously described (Zawilak et al., 2001; Donczew et al., 2015) with minor modifications. The IRD-700-labeled Bd*oriC* fragment (12 fmol, 623 bp, PCR amplified using primers P-5/P-4 and the pOC*BdoriC* plasmid as the template) and an IRD-700-labeled control DNA fragment (12 fmol, 620 bp, PCR-amplified using primers P-5/P-14 and pOC*Bd2045* as the template) were incubated with recombinant DnaA proteins of *B. bacteriovorus* (BdDnaA), *E. coli* (EcDnaA), and *Pseudomonas putida* (PpDnaA) at 30°C for 20 min in Marians’ binding buffer [20 mM HEPES/KOH, pH 8.0, 5 mM magnesium acetate, 1 mM EDTA, 4 mM DTT, 0.2% Triton X-100, 100 μM ATP, and 100 μg ml^-1^ bovine serum albumin (BSA)]. The reaction was carried out in the presence of a non-specific competitor [poly(dA-dC)•poly(dG-dT), 50 ng; Sigma, P0307]. The formed complexes were chilled on ice for 2 min and separated by electrophoresis (5 V/cm) on 4% polyacrylamide gels in 0.5× TBE (89 mM Tris, 89 mM borate, 1 mM EDTA) at 20°C. The gels were analyzed using an Odyssey CLx Infrared Imaging System and the Image Studio software (Li-Core Biosciences).

### Surface Plasmon Resonance (SPR)

For surface plasmon resonance (SPR) analysis, a 652-bp Bd*oriC* fragment was PCR amplified with biotinylated primer P-6 and non-biotinylated primer P-3, and immobilized on the chip surface (Sensor Chip SA) in a BIAcore T200 apparatus. Approximately, 100 response units (RUs) of DNA were immobilized. A non-DnaA-box DNA fragment (649 bp, PCR amplified using primers P-6 and P-13) was used as a negative control. Measurements were performed in HKM buffer (25 mM HEPES, pH 7.6, 100 mM potassium acetate, 1 mM magnesium acetate, 0.005% Tween 20) (Pei et al., 2007) in the presence of the DNA competitor, poly(dA-dC)•poly(dG-dT) (final concentration, 50 μg/ml) at a continuous flow rate of 15 μl min^-1^. At the end of each cycle (180 s association followed by 90 s dissociation), the bound proteins were removed by washing with 0.05% (w/v) SDS for 20 s, and the flow channels were equilibrated with HKM buffer until the baseline was stable. The data were analyzed using the BIA evaluation 3.0 software program.

### P1 Nuclease Assay

The P1 nuclease assay was performed as previously described (Donczew et al., 2012). The pOC*BdoriC* plasmid (112 pmol) was incubated with DnaA proteins (0, 17.5, 35, 70, and 140 pmol from *B. bacteriovorus, E. coli*, or *P. putida*), and the presence of unwound DNA was examined by P1 treatment followed by digestion with SspI. The digestion products were visualized on a 1% agarose gel using a Molecular Imager^®^ Gel Doc^TM^ XR+ System and the Image Lab Software (Bio-Rad).

### *oriC* Activity

The *E. coli* strains, WM1785 and its *polA* derivative, WM1838 (*polA^-^*, *fadA*::Tnl0), were used as host strains in the *ori* assay (Woelker and Messer, 1993). Chemically competent WM1785 and WM1838 cells were heat-shock-transformed using 50 ng of the appropriate plasmid (pBR322, pOC170, pBR322*BdoriC*, or pOC*BdoriCΔori*). The transformed cells were then cultivated on agar plates with tetracycline (12.5 μg/ml; for pBR322 and pBR322*BdoriC*) or ampicillin (100 μg/ml; for pOC170 and pOC*BdoriCΔori*) overnight at 30°C (for WM1838) or 37°C (for WM1785).

### DMS Footprinting and PE Analysis

DNA modification with dimethyl sulfate (DMS) was performed as previously described (Sasse-Dwight and Gralla, 1991; Donczew et al., 2015). The reaction mixtures (50 μl) contained 25 mM HEPES/KOH, pH 7.6, 12% (v/v) glycerol, 1 mM CaCl_2_, 0.2 mM EDTA, 5 mM ATP, 0.1 mg/ml BSA, 15 nM pOC*BdoriC*, and 6HisBdDnaA protein (0, 200, 400, 800, or 1600 nM). After the mixtures were incubated at 30°C for 10 min, 3.6 μl of 150 mM DMS (Sigma) was added to a final concentration of 10 mM, and the incubation was continued for 5 min. The reaction was quenched by the addition of 100 μl of cold Stop Buffer (3 M ammonium acetate, 1 M 2-mercaptoethanol, 20 mM EDTA). The samples were precipitated with cold ethanol, dried, dissolved in 100 μl of 1 M piperidine, and incubated at 90°C for 30 min. DNA was purified by gel filtration on Sephacryl S500 (Sigma) spin columns equilibrated in molecular-grade water. The DMS modification pattern was monitored by PE [primer extension (PE)] analysis using primers P-7, P-8, P-9, and P-10. For each PE reaction, 0.3 units of Taq DNA polymerase (Thermo Scientific), 20 fmol of DNA template, and 350 fmol of ^32^P-labeled primer were used. PE was performed using 30 cycles of 30 s at 95°C, 30 s at 55°C, and 60 s at 72°C. The samples were then separated on a 8% polyacrylamide gel under denaturing conditions and scanned with a Typhoon 8600 Variable Mode Imager (GE Healthcare).

### RIP Mapping

Replication initiation point (RIP) mapping was performed essentially as previously described (Gerbi and Bielinsky, 1997; Bielinsky and Gerbi, 1999; Matsunaga et al., 2003; Donczew et al., 2012). *B. bacteriovorus* cells were grown in 1000 ml HEPES buffer supplemented with *P. putida* cells (OD_600_ = 1.0), and *P. putida* cells were grown in LB medium (OD_600_ = 1.0). *B. bacteriovorus* were grown until the solution became viscous and slightly clear, whereupon the medium was passed through a 0.45-μm filter and then pelleted. The bacterial pellets were resuspended in 30 ml of TEN buffer (50 mM Tris-HCl, pH 8.0, 50 mM EDTA, 100 mM NaCl) and disrupted by the addition of sodium dodecyl sulfate (SDS) and sodium sarcosyl (final concentration, 1% each). The mixture was subjected to three-step extraction with phenol:chloroform:isoamyl alcohol (25:24:1, v/v) and after that 1.1 g/ml CsCl and 6 μl Midori Green Advanced DNA Stain (Nippon) were added to the aqueous phases. The genomic DNA was purified by CsCl gradient ultracentrifugation. To enrich the replication intermediates, the total isolated DNAs (75 μg for *B. bacteriovorus* and 426 μg for *P. putida*) were passed through BND-cellulose columns (Sigma-Aldrich) pre-equilibrated with NET buffer (10 mM Tris-HCl, pH 8.0, 1 mM EDTA and 1 M NaCl). The columns were washed with five volumes of NET buffer, and DNA was eluted at 50°C with NET buffer containing 1.8% caffeine. To remove nicked DNA, the recovered DNAs (48 μg for *B. bacteriovorus* and 58 μg for *P. putida*) were subjected to phosphorylation by T4 kinase (Thermo Scientific) followed by λ-exonuclease (Thermo Scientific) digestion. The PE reactions contained 1 unit of vent (exo-) DNA polymerase (Thermo Scientific), 0.6 μg of prepared DNA, and 350 fmol of ^32^P-labeled primer P-19. After 35 cycles of reaction (30 s at 95°C, 30 s at 55°C, and 60 s at 72°C), the amplified products were separated on an 8% polyacrylamide gel under denaturing conditions and analyzed with a Typhoon FLA 9500 Biomolecular Imager (GE Healthcare).

### Immunoprecipitation Assay

Immunoprecipitation assays were performed as described elsewhere (Jakimowicz et al., 2002). Briefly, *B. bacteriovorus* HI cells were grown to OD = 1.0 in 40 ml of PYE medium, and then formaldehyde was add to final concentration 1% (v/v) and the samples were incubated for 30 min. Anti-6HisBdDnaA polyclonal antibodies (ProteoGenix) were used to precipitate BdDnaA-DNA nucleoprotein complexes, and PCR was used to amplify regions of interest (primers P-3/P-4 for *oriC* and primers P-11/P-12 for non-box DNA). The PCR fragments were resolved on 1.5% agarose gels and analyzed using a Gel Doc^TM^ XR+ Imaging System (Bio Rad).

## Results

### The *In silico*-Predicted Origin of Replication for *B. bacteriovorus* Lies within the Conserved Gene Cluster of *rnpA-rpmH-dnaA-dnaN-recF-gyrB-gyrA*

To identify the *oriC* of *B. bacteriovorus*, we employed different *in silico* tools, including analysis of gene arrangement, GC-skew analysis, identification of DnaA boxes, and the WebSIDD tool (SIDD, stress-induced DNA duplex destabilization) for localizing the DNA-unwinding element (DUE) (for details see Materials and Methods). Similar to the previous findings of Gao et al. (2013), we obtained predictions for *oriC*-type replication origins within the ∼250-bp-long *dnaA-dnaN* intergenic regions of the genomes of *B. bacteriovorus* (HD100 and str. Tiberius strains) and other species belonging to this genus, including *Halobacteriovorax marinus* SJ and *B. exovorus* JSS (*B. exovorus* JSS is not yet included in the DoriC data set created by Gao et al., 2013). The predicted *Bdellovibrio oriC* region contains eight putative DnaA boxes (see Supplementary Figure S1; **Figure 6**). Interestingly, two of the putative DnaA boxes (boxes 7 and 8; Supplementary Figure S1; **Figure 6**) are unusually situated within the *dnaA* gene, at its 3′-end. The position of the DUE could be readily derived from the SIDD plots generated for all of the analyzed organisms, with the exception of *B. exovorus* JSS. As seen for other bacterial origins, the first in the cluster of DnaA boxes could be assigned to a position approximately two helical turns distant from the border of a strong SIDD site (see Supplementary Figure S1; **Figure 6**). In the case of *B. exovorus* JSS, we were able to assign a “predicted *oriC*” based on the high similarity of DnaA box distances and orientations in this organism and the two *B. bacteriovorus* strains for which we were able to predict *oriC*s. In all four genomes, the predicted *oriC* is flanked upstream by the *dnaA*, *rpmH*, *rnpA*, and *yidC* (*oxaA*) genes, and downstream by the *dnaN*, *recF*, *gyrB*, and *gyrA* genes. This particular *oriC* gene context is also found in many genomes from the Actinobacteria and Firmicutes (Ogasawara et al., 1985).

In sum, the *in silico*-predicted *B. bacteriovorus oriC* region contains origin-characteristic elements (DnaA boxes and a DUE) and is located between the *dnaA* and *dnaN* genes within the gene cluster of *rnpA-rpmH-dnaA-dnaN-recF-gyrB-gyrA*, which is conserved in some bacterial species.

### *B. bacteriovorus oriC* is Specifically Bound by BdDnaA *In vivo* and *In vitro*

**FIGURE 1:**
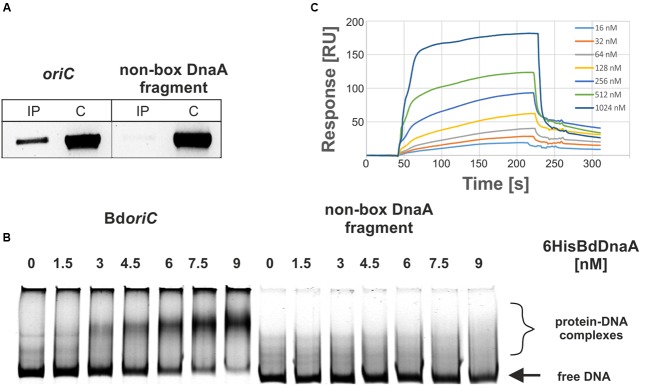
**BdDnaA interacts specifically with Bd*oriC in vivo* and *in vitro.* (A)** *In vivo* identification of the BdDnaA-Bd*oriC* complexes. Anti-6HisBdDnaA polyclonal antibodies were used to immunoprecipitate BdDnaA-Bd*oriC* complexes cross-linked with formaldehyde. The *oriC* region and non-box DnaA fragment (negative control) were amplified by PCR (see Materials and Methods). Lanes: IP, immunoprecipitated DNA; and C, total genomic DNA extracted from *B. bacteriovorus* cells (PCR control). **(B)** EMSA analysis of the interaction between 6HisBdDnaA and Bd*oriC in vitro*. IRD-700 labeled DNA fragments, Bd*oriC* (635 bp), and control DNA (part of the *bd2045* gene, 620 bp) were incubated with increasing amounts of 6HisBdDnaA proteins, and the formed nucleoprotein complexes were analyzed on a 4% polyacrylamide gel. **(C)** SPR analysis of the interaction between 6HisBdDnaA and Bd*oriC in vitro*. Biotinylated versions of the Bd*oriC* region (652 bp) and a non-box DNA fragment (649 bp) were immobilized on the chip surface (Sensor Chip SA) in a Biacore T200 apparatus. Sensograms were obtained for different concentrations of 6HisBdDnaA interacting with the DNA fragment containing Bd*oriC*. The BIA evaluation 3.0 software was used for data analysis.

To determine whether the *in silico*-predicted *B. bacteriovorus oriC* region is bound *in vivo* by the initiator BdDnaA protein, we performed immunoprecipitation assays using antibodies against the purified 6HisBdDnaA protein (Supplementary Figure S2). The formaldehyde cross-linked BdDnaA-Bd*oriC* complexes formed in *B. bacteriovorus* grown under host-independent conditions were enriched by affinity chromatography, and the released DNA fragments were identified by PCR (**Figure 1A**). We obtained strong PCR signals using primers for the Bd*oriC* fragment but not a non-box DnaA fragment (**Figure 1A**), indicating that BdDnaA-Bd*oriC* complexes were successfully detected.

To examine whether the BdDnaA protein interacts with Bd*oriC in vitro*, we applied electrophoretic mobility shift assays (EMSAs) and SPR. Our EMSAs demonstrated that the Bd*oriC* region, but not the non-DnaA box fragment, was bound specifically by the BdDnaA protein (**Figure 1B**). Interestingly, although the Bd*oriC* contains eight putative DnaA boxes, only one nucleoprotein complex was observed. Moreover, increasing the protein concentration did not lead to the formation of additional higher-molecular-weight complexes. The interaction between BdDnaA and Bd*oriC* was also confirmed by SPR analysis (**Figure 1C**), which showed that the RU values were proportional to the BdDnaA concentration.

Together, these findings indicate that the *in silico*-predicted *B. bacteriovorus oriC* is bound specifically by the initiator protein, BdDnaA, *in vitro* and *in vivo.*

### The BdDnaA Protein Specifically Binds DNA Sequences Corresponding to the *In silico*-Assigned DnaA Boxes

**FIGURE 2:**
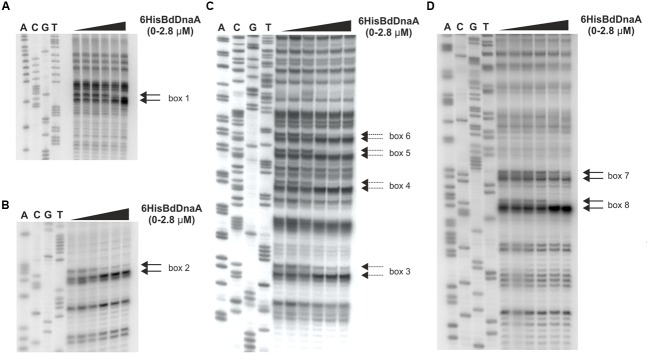
**6HisBdDnaA recognizes specific DNA sequences within Bd*oriC*.** DMS footprinting. pOC*BdoriC* plasmids were incubated with increasing amounts of 6HisBdDnaA proteins (0, 0.175, 0.35, 0.7, 1.4, and 2.8 μM), treated with DMS, and then used as a substrate for primer extension (PE) analysis. **(A–D)** DMS footprints were obtained with ^32^P-labeled primers P-10 **(A)**, P-9 **(B)**, P-18 **(C)**, and P-7 **(D)**. Primers P-7, P-9, and P-10 are complementary to the lower strand, while primer P-18 is complementary to the upper strand. Solid lines and dashed arrows indicate the nucleotides of the lower strand and upper strand, respectively, that become sensitive to DMS upon protein binding.

To further define the *in silico*-predicted DnaA boxes and gain additional insight into their abilities to bind the BdDnaA protein, we applied footprinting experiments using DMS. This agent primarily methylates deoxyguanosine residues, making the proximate phosphodiester bond susceptible to piperidine cleavage (Maxam and Gilbert, 1977). The pOC*BdoriC* plasmid, which contained the entire *oriC* region, was incubated with increasing concentrations of BdDnaA protein, and then subjected to DMS modification and subsequent PE of piperidine-cleaved DNA. We identified 16 nucleotides that exhibited BdDnaA-dependent protection from DMS modification: eight Gs on the upper strand and eight Gs on the lower strand (**Figure 2**). Fourteen of them are located within the seven of eight *in silico*-predicted DnaA boxes, while the remaining two lie between *in silico*-predicted DnaA boxes (box; **Figure 2** and see also **Figure 6**). Thus, the DMS footprinting confirmed the results of our *in silico* analysis and allowed us to identify additional DnaA-binding sites. Moreover, this analysis showed that the two unusually located DnaA boxes, boxes 7 and 8 (**Figure 6A**), are bound by the BdDnaA protein (**Figure 2D**).

Based on the assumption that, as in other bacteria, the DnaA box sequence of *B. bacteriovorus* consists of nine nucleotides, we aligned the DNA sequences in the vicinity of the protected nucleotides and obtained a proposed consensus sequence for the BdDnaA-binding motif, 5′-NN(A/T)TCCACA-3′, which we designated the DnaA box (**Figure 6B**; Supplementary Table S2).

Collectively, these analyses show that BdDnaA specifically binds to eight sites (**Figures 2A–D**) within the identified Bd*oriC* region of *B. bacteriovorus*.

### DnaA Proteins from Prey Organisms Specifically Bind Bd*oriC In vitro*

**FIGURE 3:**
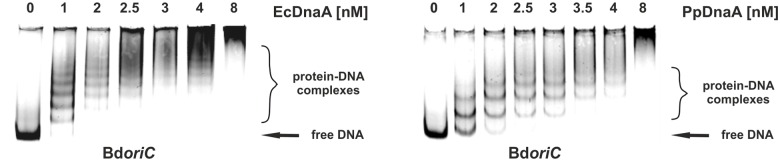
**DnaA proteins from host organisms, *Escherichia coli* and *P. putida*, interact specifically with *B. bacteriovorus oriC.*** EMSA – an IRD-700-labeled Bd*oriC* fragment (635 bp) was incubated with increasing amounts of DnaA proteins from *E. coli* (EcDnaA) or *P. putida* (PpDnaA), and the nucleoprotein complexes were analyzed on a 4% polyacrylamide gel.

Interestingly, the binding mode of the *E. coli* DnaA protein to the *oriC* of *E. coli* (Ec*oriC*) (Weigel et al., 1997) appears to differ from the interaction between *B. bacteriovorus* BdDnaA and the Bd*oriC.* In contrast to *B. bacteriovorus*, in which only a single nucleoprotein complex was formed (**Figure 1B**), the interaction of *E. coli* and *P. putida* DnaAs with the Bd*oriC* region yielded multiple discrete nucleoprotein complexes that formed a ladder of retarded bands on the gel indicating that the DnaA boxes were sequentially bound by the DnaA proteins (**Figure 3**). This difference in orisome formation prompted us to question how the arrangement of DnaA boxes and/or the properties of DnaA influence the formation of nucleoprotein complexes, and whether the formation of a single BdDnaA-Bd*oriC* complex is specific to *B. bacteriovorus*. To answer these interesting questions, we analyzed orisome formation in heterologous systems. The Bd*oriC* region from *B. bacteriovorus* was incubated with DnaA proteins from prey organisms (*E. coli* or *P. putida*) and the formed nucleoprotein complexes were analyzed using EMSAs. In these *in vitro* heterologous systems (as in the homologous system; **Figure 1B**), the observed nucleoprotein complexes formed in a protein-concentration-dependent manner (**Figure 3**). Surprisingly, the DnaA proteins of the prey organisms exhibited higher affinities toward the *B. bacteriovorus oriC* than the BdDnaA from *B. bacteriovorus* toward its own Bd*oriC* region. The Bd*oriC* region from *B. bacteriovorus* was almost completely bound at the lowest tested concentration of DnaA from *E. coli* and *P. putida* (1 nM; **Figure 3**), whereas in the homologous system, the nucleoprotein complex was detectable only at *B. bacteriovorus* DnaA concentrations >3 nM (**Figure 1B**). As the concentrations of *E. coli* or *P. putida* DnaA proteins increased, the complexity of the band pattern increased until a critical point was reached, whereupon the ladder pattern was replaced by diffuse, highly retarded bands indicative of large complexes (**Figure 3**). Thus, whereas the *B. bacteriovorus* DnaA appears to exhibit a unique binding mode to the *B. bacteriovorus oriC*, the DnaA proteins of prey organisms bind their own and *B. bacteriovorus oriC* regions in a similar manner. We also observed that the *oriC* region from *B. bacteriovorus* was not specifically bound by DnaA proteins from non-prey organisms (e.g., *Streptomyces coelicolor*; data not shown).

Together, the results of *in vitro* analysis indicate that DnaA proteins from prey bacteria bind the Bd*oriC* region specifically and with a high affinity, forming multiple nucleoprotein complexes.

### DNA Unwinding Takes Place at the 5′-end of Bd*oriC*

**FIGURE 4:**
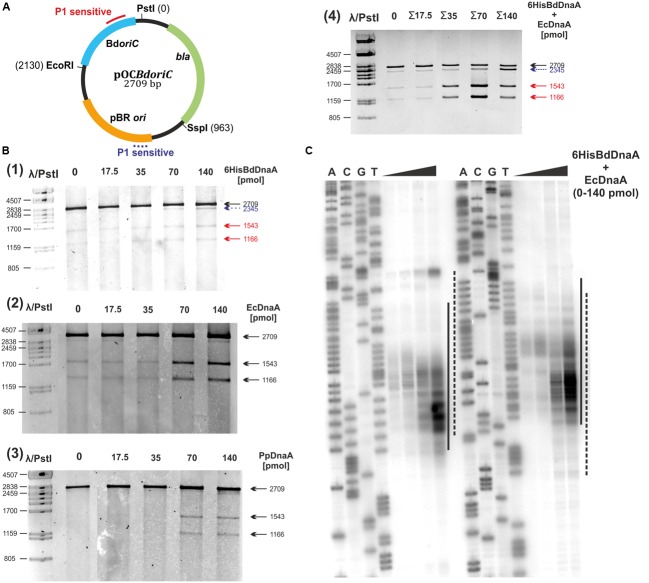
**6HisBdDnaA unwinds DNA within the Bd*oriC in vitro*. (A–C)** *In vitro* identification of the DUE in the *B. bacteriovorus oriC* region. **(A)** Map of the plasmid used in the P1 nuclease assay. The Bd*oriC* region, the plasmid origin of replication, and the positions of the most important restriction sites are marked. Solid and dashed lines indicate P1-sensitive sites for BdDnaA-dependent and BdDnaA-independent unwinding, respectively. **(B)** P1 nuclease assay localizing the region unwound by 6HisBdDnaA. pOC*BdoriC* was incubated with increasing amounts of the 6HisBdDnaA (1), EcDnaA (2), PpDnaA (3) or a 1:1 (molar ratio) mixture of 6HisBdDnaA and EcDnaA (4). The plasmid was then treated with P1 nuclease and cut with SspI, and the resulting DNA fragments were analyzed by separation on a 1% agarose gel. **(C)** Determination of the *B. bacteriovorus oriC* sequence unwound by the mixture of 6HisBdDnaA and EcDnaA *in vitro*. pOC*BdoriC* was incubated with increasing amounts of mixed 6HisBdDnaA and EcDnaA (1:1 molar ratio), treated with P1 nuclease, and cut with SspI. The generated DNA fragments were used as the substrate for PE analysis. The ^32^P-labeled primers, P-10 and P-17, were complementary to the non-coding strand (with respect to the *dnaA* gene; left panel) and coding strand (right panel), respectively, and they were also used for sequencing reactions (sequenced bases A, C, G, T). Dashed lines correspond to the *in silico*-identified DUE, while solid lines indicate the P1-nuclease-sensitive sites of pOC*BdoriC*.

To experimentally verify the *in silico*-predicted *B. bacteriovorus* DUE within Bd*oriC*, we used a P1 nuclease assay. Supercoiled pOC*BdoriC* plasmids containing all of the predicted DnaA boxes (Supplementary Table S1) were incubated with increasing amounts of BdDnaA and subsequently treated with P1 nuclease, which hydrolyzes single-stranded DNA at the opened helix and hence linearizes the unwound plasmid (Donczew et al., 2012). Subsequent digestion with SspI allowed us to approximate the region unwound by BdDnaA. We detected faint bands providing evidence for DnaA-dependent DNA unwinding; the observed DNA fragments were ∼1.2 and 1.5 kb, indicating that the P1 hydrolysis site corresponded to the *in silico*-predicted DUE of *B. bacteriovorus* (**Figure 4B1**). We also observed an additional DNA fragment of about 2.3 kb (**Figure 4A**), likely corresponding to a DnaA-independent P1-sensitive site located within the plasmid origin of pBR322 (pOC*BdoriC*). It was previously suggested that the *ori* of pBR322 contains a helically unstable region (Kowalski et al., 1988; Donczew et al., 2012).

Since EcDnaA and PpDnaA strongly bound Bd*oriC*, we examined whether these prey proteins could unwind Bd*oriC*. Surprisingly, P1- and SspI-mediated digestion generated the same patterns obtained using BdDnaA (**Figure 4B2,3**), indicating that both proteins could *in vitro* unwind DNA within the Bd*oriC* region. The efficiency of DNA unwinding was much higher for the prey proteins (particularly that of *E. coli*) than for BdDnaA (**Figure 4B2,3**). Moreover, when we performed P1 assays with an equimolar mixture of DnaA proteins from *B. bacteriovorus* and *E. coli*, strong bands were observed at a minimal protein concentration (35 nM; **Figure 4B4**) that was even lower than that found to yield a similar result with the *E. coli* protein alone (70 nM; **Figure 4B2**).

**FIGURE 5:**
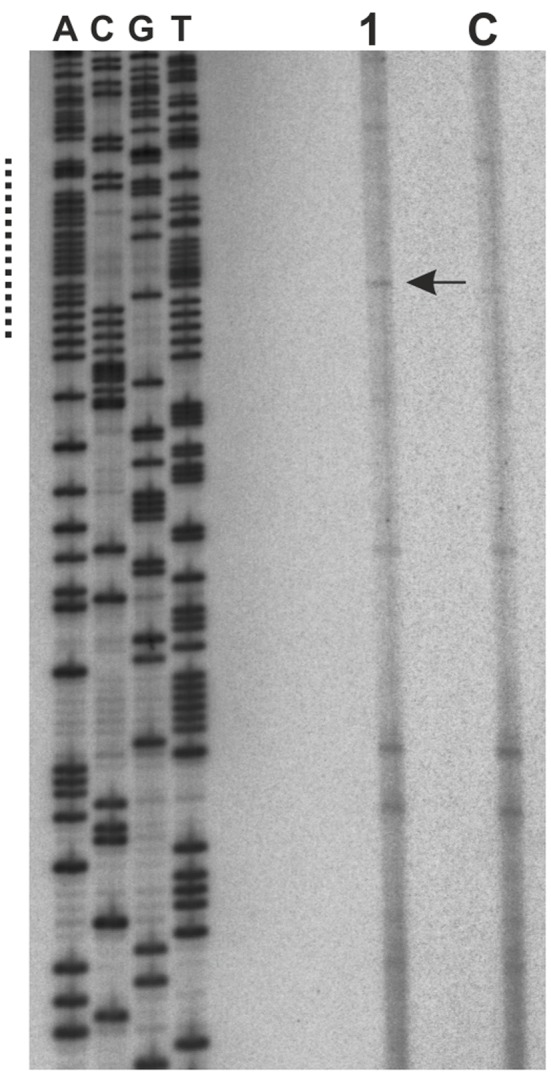
**The replication initiation point is located within the Bd*oriC* DUE.** Enriched replication intermediates were used as the substrate for PE analysis. ^32^P-labeled primer P-19 was complementary to the non-coding strand (with respect to the *dnaA* gene) and was also used for sequencing (A, C, G, T) the pOC*BdoriC* plasmid DNA. The dotted line corresponds to the *in vitro*-identified DUE. Line 1 represents *B. bacteriovorus* grown on *P. putida*, while line C represents *P. putida* (control). Arrow indicates the transition point between continuous and discontinuous DNA synthesis.

**FIGURE 6:**
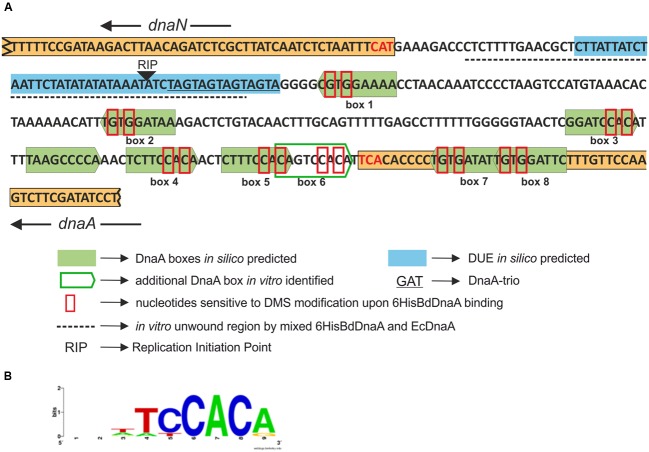
**Organization of the *B. bacteriovorus oriC* region. (A)** The results from our *in silico*, *in vitro* and *in vivo* analyses. **(B)** Consensus sequence of the *B. bacteriovorus* DnaA box, as identified using an online tool WebLogo (Crooks et al., 2004).

To precisely map the unwound region, we performed PE of P1-cleaved pOC*BdoriC* plasmids using Taq polymerase and ^32^P-labeled primers flanking the *in silico*-predicted DUE (for details see Materials and Methods and Supplementary Table S1). Since BdDnaA alone yielded only faint signals in the P1 assay, we used an equimolar mixture of *B. bacteriovorus* and *E. coli* proteins (**Figure 4C**). The observed extension products confirmed that DNA unwinding occurs within the *in silico*-predicted DUE sequence at the 5′-end of the *oriC* region, and allowed us to estimate the unwound region as spanning ∼55 bp (**Figures 4C** and **6A**). Moreover, RIP mapping showed that Bd*oriC* is the replication initiation site *in vivo* (**Figure 5**).

Together, these results show that Bd*oriC* is unwound at the 5′-end by its own BdDnaA, as well as by DnaA proteins from the prey species, *E. coli* and *P. putida.*

### Bd*oriC* Is Not Able to Initiate DNA Replication in *E. coli*

Since Bd*oriC* was specifically bound and unwound by the DnaA from *E. coli*, we next tested whether the predator’s *oriC* could initiate replication in its prey. To investigate ability of Bd*oriC* to promote replication in *E. coli*, we performed a set of heterologous transformations in which the pBR322 plasmid (a negative control) and its derivatives carrying Bd*oriC* or Ec*oriC* (a positive control) regions (Supplementary Table S1) were assayed for *oriC*-dependent initiation of replication in the *E. coli polA^-^* strain (Langer et al., 1996; Zawilak-Pawlik et al., 2005). ColE1-type plasmids (such as pBR322) require DNA polymerase I for their replication; thus, only a construct containing a functional *oriC* region (and conferring pBR322-encoded ampicillin resistance, Amp^R^) can replicate in the absence of DNA polymerase I (such as found in *E. coli polA^-^*). Among the analyzed constructs, only the pOC170 plasmid carrying the Ec*oriC* was replicative (**Table 1**), suggesting that the Bd*oriC* region does not promote the initiation of replication in *E. coli*. Indeed, no plasmid containing the Bd*oriC* region yielded ampicillin-resistant transformants in the *polA*-deficient strain, even after prolonged incubation.

**Table 1:** Replication activity of the BdoriC region in *Escherichia coli.*

	Transformation efficiency in *E. coli* (number of transformants per μg of DNA)	
Plasmid	WM 1838 polA- (30°C)	WM 1785 polA+ (37°C)
pBR322_BdoriC	0	4.79 × 10^4^
	0	4.3 × 10^4^
*pOC170 (EcoriC)*	8.6 × 10^4^	9.7 × 10^4^
	7.9 × 10^4^	9.3 × 10^4^
*pBR322*	0	12.8 × 10^4^
	0	11.0 × 10^4^
*pOCBdoriCΔEcori*	0	8.8 × 10^4^
	0	7.6 × 10^4^


Together, these results show that *B. bacteriovorus oriC* is not a substrate for replication in its prey organisms.

## Discussion

*Bdellovibrio bacteriovorus* is a small predatory bacterium that exhibits a peculiar biphasic life cycle during which two different types of cells are produced: non-replicating highly motile cells (the free-living phase) and replicating cells (the intracellular-growth phase) (Starr, 1975). The process of chromosomal replication in *B. bacteriovorus* must therefore be temporally and spatially regulated to ensure that it is coordinated with cell differentiation and cell cycle progression. Although, we know that chromosomal replication in bacteria is mainly regulated at the initiation step, nothing is known about this process in *B. bacteriovorus*. Here, we report the first characterization of key elements of replication initiation, namely the BdDnaA protein and the Bd*oriC* region, in a bacterium that preys on other bacteria. Surprisingly, we show that DnaA proteins from prey bacteria specifically bind and unwind the *oriC* region of their predator.

We identified the *B. bacteriovorus oriC* region within the *rnpA-rpmH-dnaA-dnaN-recF-gyrB-gyrA* gene cluster, which is conserved even in distantly related bacterial species (Ogasawara and Yoshikawa, 1992). Bd*oriC* is localized between the *dnaA* and *dnaN* genes and is relatively small (the intergenic region is 232-bp long). Our immunoprecipitation assays demonstrated that *oriC* is specifically bound by the BdDnaA *in vivo*. Eight DnaA-binding motifs were identified using EMSA and DMS footprinting. Six are typically located within the intergenic region, while the remaining two (boxes 7 and 8; **Figure 2A**) exhibit an unusual localization in the 3′-end of the *dnaA* gene (before the stop codon). This raises an interesting question regarding the roles of these atypical located boxes in the initiation of chromosomal replication: are they involved in orisome formation (e.g., by serving as a scaffold for DnaA protein oligomerization) and/or do they contribute to regulating the frequency of initiation? Future *in vivo* studies will be needed to elucidate the role of these boxes in the initiation of chromosomal replication in *B. bacteriovorus*.

Sequence comparison of the DnaA-binding sites (**Figure 6B**; Supplementary Table S2) enabled us to propose a consensus sequence for the *B. bacteriovorus* DnaA box. This sequence, 5′-NN(A/T)TCCACA-3′, is similar to the so-called “perfect” box sequence (i.e., that which binds DnaA with the highest affinity) of *E. coli* (TTATCCACA). Sequence analysis of high-affinity DnaA boxes from various bacteria, including low-GC (*Helicobacter pylori*, TCATTCACA) and high-GC [*Streptomyces*, TT(G/C)TCCACA] organisms (Wolański et al., 2015), revealed that the 4th, 6th, 7th, 8th, and 9th residues of these DnaA boxes are conserved in phylogenetically distant organisms. This suggests that the interaction of DnaA with these residues follows similar specificity rules in all of the tested bacterial species. Our findings are consistent with previous reports showing that these bases of the DnaA box are important for interactions between DnaA and the DnaA box (Fujikawa et al., 2003; Tsodikov and Biswas, 2011). On the other hand, the first three positions in the DnaA box sequence appear to be relatively relaxed, and are thus likely to confer the species specificity of DnaA-DNA interactions. Therefore, our findings indicate that the consensus sequence of the *B. bacteriovorus* DnaA box represents a typical eubacterial DnaA-binding motif.

The second basic functional module of the replication origin is the DUE region, which is responsible for the unwinding of DNA. We identified the DUE within the *B. bacteriovorus oriC* using three independent methods (*in silico* prediction, P1 nuclease assay and RIP; **Figures 4** and **5**; Supplementary Figure S1). As in other bacteria, such as *E. coli* (Hwang and Kornberg, 1992), *H. pylori* (Donczew et al., 2012), and *Thermoanaerobacter tengcongensis* (Pei et al., 2007), the DUE region of *B. bacteriovorus* is located proximal (∼2 helical turns) to the first DnaA box (**Figure 6**). Moreover, this DnaA box has the same DUE-relative orientation as corresponding boxes in other *oriC* regions (Wolański et al., 2015). Although the *B. bacteriovorus* DUE region is AT-rich, it does not contain the typical AT-rich 13mer repeat found in other bacteria (e.g., *E. coli* or *Bacillus subtilis*) (Rajewska et al., 2012). However, we identified an array of four DnaA-trio elements (GAT) within the DUE region; they are located near DnaA box 1, and are separated from this box by a short GC-rich region (**Figure 6A**). DnaA-trios are newly identified elements found within the DUEs of bacterial *oriC*s (Richardson et al., 2016). Recently, Richardson et al. (2016) demonstrated that these trios play an essential role in replication initiation by enabling DnaA to form a filament on single-stranded DNA, thereby promoting the unwinding of *oriC*.

The AT-rich sequences of the DUEs showed very little homology between *B. bacteriovorus* and *E. coli* or *P. putida*. Surprisingly, however, the DnaAs from these prey bacteria were found to unwind Bd*oriC* within the AT-rich region *in vitro* (**Figure 4B4**). Moreover, compared to BdDnaA, EcDnaA, and PpDnA were more efficient in opening double-stranded DNA at the DUE region of Bd*oriC*. This is particularly interesting in the case of EcDnaA, which requires HU proteins to open the *E. coli* replication origin *in vitro* (Dixon and Kornberg, 1984; Hwang and Kornberg, 1992). Our *in vitro* experiments revealed that both EcDnaA and PpDnaA can unwind Bd*oriC* in the absence of HU or any other ‘prey-derived’ protein (**Figure 4B**). This presumably indicates that DnaA proteins are intrinsically capable of unwinding a DUE once a proper DnaA oligomer has been formed. Such oligomerization depends on both the DnaA-box scaffold and the presence of additional regulatory proteins that help DnaA initiate complex formation (e.g., HU and DiaA in *E. coli*) (Hwang and Kornberg, 1992; Ishida et al., 2004; Keyamura et al., 2007). The factors responsible for stimulating this unwinding of DNA in *B. bacteriovorus* remain to be identified.

**FIGURE 7:**
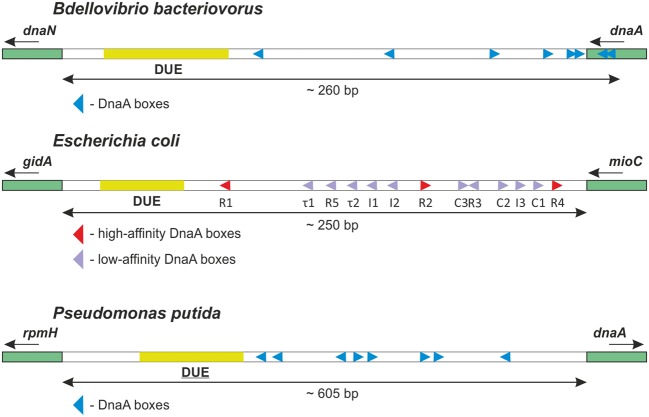
**The structures of the *oriC* regions from *B. bacteriovorus* and two of its prey bacteria.** Underlined DUE indicates experimentally unconfirmed unwinding. The direction of each triangle represents the orientation of a DnaA box. The small arrows below gene names indicate their gene orientations.

Interestingly, the *in vitro* binding mode of BdDnaA differs from those of EcDnaA and PpDnaA, despite having similar *oriC* regions structures (**Figure 7**). The binding of BdDnaA to Bd*oriC* results in the formation of a single nucleoprotein complex (**Figure 1B**), whereas the binding of EcDnaA or PpDnaA to Bd*oriC* yielded multiple discrete nucleoprotein complexes (**Figure 3**). This suggests that BdDnaA binds simultaneously at all eight boxes, whereas the DnaA proteins of the prey species sequentially bind the DnaA boxes within Bd*oriC*. Moreover, EcDnaA and PpDnaA exhibited higher affinities toward Bd*oriC* compared to BdDnaA. This further suggests that additional factors contribute to BdDnaA oligomerization/DNA binding, and thus may be involved in regulating the chromosomal replication of *B. bacteriovorus*.

It is noteworthy that the *in vitro* ability of prey’s DnaA proteins to bind and unwind Bd*oriC* region may not presumably reflect the *in vivo* situation. Growth of *B. bacteriovorus* in the periplasma of host bacteria and the production of many proteases represent some of limitations of transferring *in vitro* results to *in vivo* conditions.

Similar to another Gram-negative bacterium, *Caulobacter crescentus*, *B. bacteriovorus* exhibits a dimorphic life cycle in which replicative cells originate from non-replicative cells (Janakiraman and Bum, 2000). In *C. crescentus*, the master regulator, CtrA, temporally and spatially coordinates chromosomal replication with the developmental program by regulating the activity of *oriC* (Laub et al., 2002). In future work, we plan to identify one or more proteins that might control the initiation of chromosomal replication in *B. bacteriovorus*.

In sum, we herein identify the key elements of *B. bacteriovorus* chromosomal replication initiation, DnaA and *oriC*, and characterize their interaction *in vivo* and *in vitro*. We also show that DnaA proteins from prey bacteria bind the Bd*oriC* region specifically and with high affinity, forming multiple nucleoprotein complexes. Finally, we demonstrate that Bd*oriC* is unwound at 5′-end by its own DnaA as well as by those of the prey species, *E. coli* and *P. putida*. This work provides important new entry points toward improving our understanding of the initiation of chromosomal replication in this predatory bacterium.

## Author Contributions

LM, RD, and JZ-C designed research, LM performed *in vitro* and *in vivo* research, CW performed *in silico* research, AZ-P, RD, and CW performed critical revision, LM and JZ-C wrote the paper.

## Conflict of Interest Statement

The authors declare that the research was conducted in the absence of any commercial or financial relationships that could be construed as a potential conflict of interest.

## Footnotes

1 http://genskew.csb.univie.ac.at/

2 http://benham.genomecenter.ucdavis.edu/sibz/
