# Supplementary material for: Initiation of Chromosomal Replication in Predatory Bacterium Bdellovibrio bacteriovorus
Source: Front Microbiol. 2016 Nov 28;7:1898. doi: 10.3389/fmicb.2016.01898 (PMC5124646; doi:10.3389/fmicb.2016.01898)
Supplement: Supplementary file 1 [file Data_Sheet_1.docx]

**Supplementary Materials**

**Fig. 1S. *In silico* identification of the putative *B. bacteriovorus* *oriC* region.**

SIDD plots and DnaA box assignments for the predicted *oriC* regions of *B. bacteriovorus* HD100, *B. bacteriovorus* str. Tiberius, *B. exovorus* JSS, and *B. marinus* SJ. The Y-axis shows the free energy values calculated for individual base pairs; we used WebSIDD (Bi and Benham, 2004) (http://benham.genomecenter.ucdavis.edu/sibz/) to identify different degrees of negative superhelicity for the input sequence (pink σ, -0.06; red σ, -0.055; dark orange σ, -0.05; light orange σ, -0.045; pale orange σ, -0.04) and plotted them as a graph (values ≤0 kcal·mol^‒1^ indicate strand opening). The X-axis shows DNA sequences by position: DnaA boxes are indicated in red with the curvature indicating orientation, while flanking genes are shown as gray boxes with the gene name and direction of transcription indicated by an arrowhead. Elements are shown to scale.

**Fig. 2S Expression and affinity purification of the *B. bacteriovorus* 6HisBdDnaA protein.**

6HisBdDnaA was isolated from *E. coli* BL21 containing the pET28a(+)*BddnaA* plasmid. Proteins were resolved by 10% SDS-PAGE and stained with Coomassie brilliant blue. Lanes: M, Pierce™ Unstained Protein Molecular Weight Marker; 1, uninduced cell extract; 2, IPTG-induced cell extract (1 mM IPTG, 3 h); 3, cell lysate after sonication; 4, soluble cell lysate after sonication and centrifugation; 5, proteins bound to the affinity column; 6, proteins not bound to the affinity column; and 7-9, proteins eluted with LG_100_ buffer containing 100 mM imidazole.

**Table 1S.** Primers and plasmids

| **Primers** | **5’ – 3’ sequence** | |
| --- | --- | --- |
| P-1 | TACGGATCCATGGAATTAAACTCGTCTTTTTGGACAC | |
| P-2 | AATCTCGAGCTCTTCCACAACTCTTTCCACAGTCC | |
| P-3 | ACCGAATTCCCTGGATAAATCTTTGGTGGATATCGG | |
| P-4 | GCACTGCAGGATCTGATCAGTAAGGCTGACTTCC | |
| P-5 | IRD700-CATCGATAGGATATCCTGGG | |
| P-6 | Biotin- CATGTTTGACAGCTTATCATCG | |
| P-7 | CTGGAACGGGTCAAGAATCTAC | |
| P-8 | AACATTTGTGGATAAAGACTC | |
| P-9 | AATGTGGATCCGAGTTACCC | |
| P-10 | CTGCAAAGTTGTACAGAGTC | |
| P-11 | ACCGAATTCCATCATCATGACCGATGCCG | |
| P-12 | AGGCTGCAGCGGTGTAGATGTCAGCATAC | |
| P-13 | ACGGAATTCGCTTCAGATCAAACATGTAAGACGCC | |
| P-14 | ACGCTGCAGGAGCACGCATTGATCGAATATGTGGC | |
| P-15 | GCACTGCAGCCTGGATAAATCTTTGGTGGATATCGG | |
| P-16 | ACTGAATTCGATCTGATCAGTAAGGCTGACTTCC | |
| P-17 | GCTTCTCAACGATGTTCTGTG | |
| P-18 | CATGTTTGACAGCTTATCATCG | |
| P-19 | TTACATGGACTTAGGGGATTTGTTAGG | |
|  |  | |
| **Plasmids** | **Feature** | **Reference/source** |
|  |  |  |
| pOC170 | Plasmid carrying the *E. coli* oriC sequence, the replication origin of pBR322 on the NotI cassette and the *bla* gene of pBR322. | (Messer et al., 1992) |
| pOC*BdoriC* | A pOC170 derivative, lacking *E. coli* *oriC*, containing Bd*oriC* region amplified with primers P-3 and P-4 and cloned into PstI/EcoRI sites. | This work |
| pOC*Bd2045* | A pOC170 derivative, lacking *E. coli* *oriC*, containing DNA fragment of *B. bacteriovorus* *bd2045* gene amplified with primers P-13 and P-14 and cloned into PstI/EcoRI sites. | This work |
| pOC*BdoriCΔori* | A pOC*BdoriC* derivative, lacking plasmids *ori*. | This work |
| pBR322*BdoriC* | A pBR322 derivate containing Bd*oriC* region amplified with primers P-15 and P-16 and cloned into PstI/EcoRI sites. | This work |
| pET28a(+)*BddnaA* | A pET28a(+) derivate containing *dnaA*. | This work |
|  |  |  |

**Table 2S Sequences of BdDnaA-boxes within *B. bacteriovorus oriC***

| **BdDnaA-box** | 5’ – 3’ sequence | | | | | | | | |
| --- | --- | --- | --- | --- | --- | --- | --- | --- | --- |
| box 1 | T | T | T | T | C | C | A | C | G |
| box 2 | T | T | A | T | C | C | A | C | A |
| box 3 | G | G | A | T | C | C | A | C | A |
| box 4 | T | C | T | T | C | C | A | C | A |
| box 5 | C | T | T | T | C | C | A | C | A |
| box 6 | C | A | G | T | C | C | A | C | A |
| box 7 | A | A | T | A | T | C | A | C | A |
| box 8 | G | A | A | T | C | C | A | C | A |
| consensus | **N** | **N** | **(A/T)** | **T** | **C** | **C** | **A** | **C** | **A** |
