# Supplementary figures and images for: Initiation of Chromosomal Replication in Predatory Bacterium Bdellovibrio bacteriovorus
Source: Front Microbiol. 2016 Nov 28;7:1898. doi: 10.3389/fmicb.2016.01898 (PMC5124646; doi:10.3389/fmicb.2016.01898)

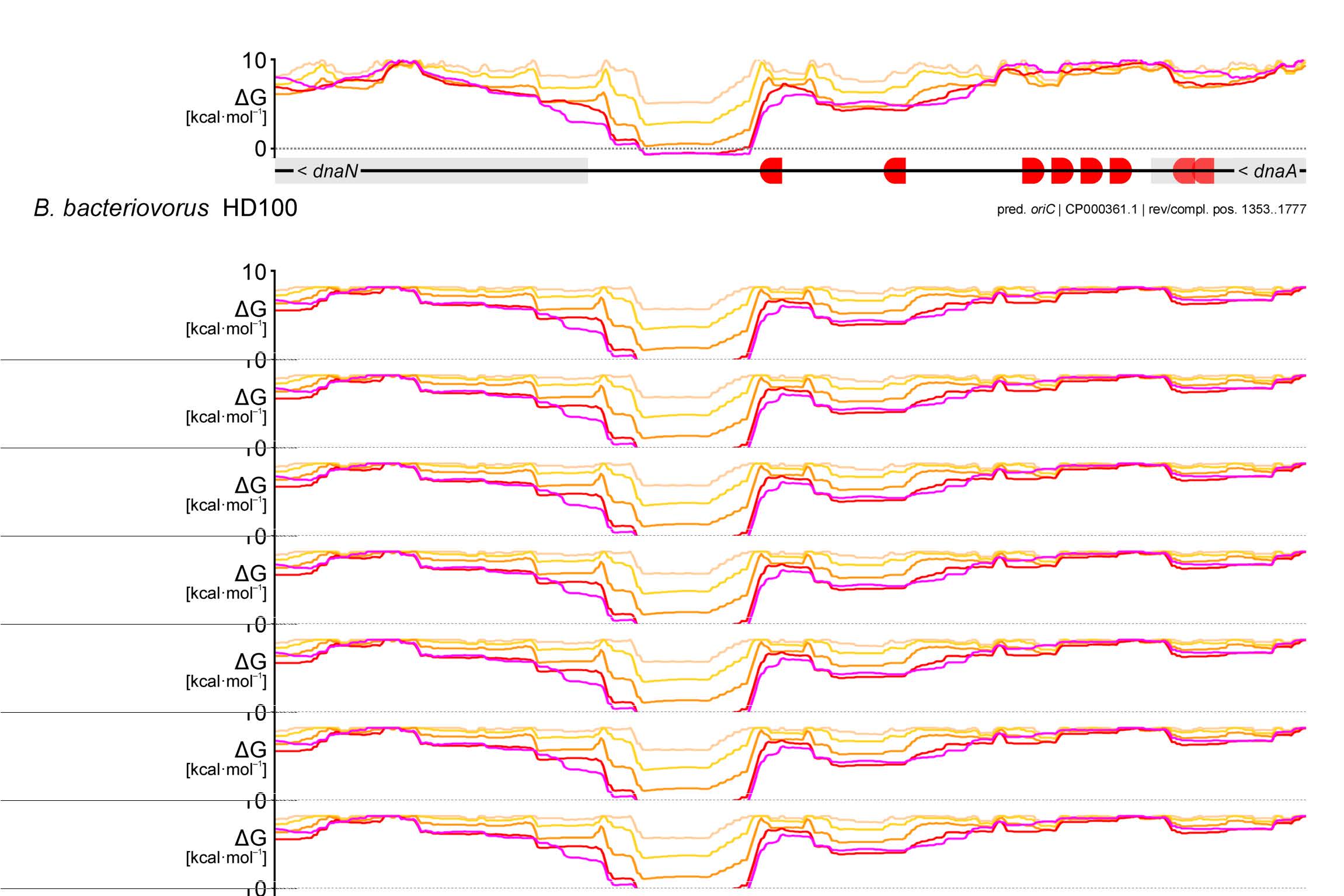

Supplement: Supplementary file 2 [file Image_1.jpg]

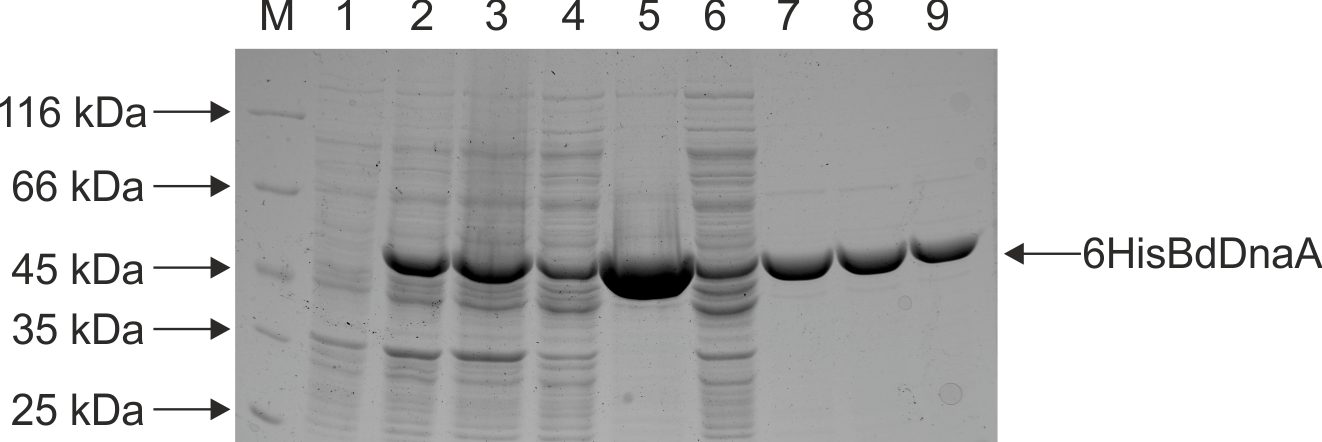

Supplement: Supplementary file 3 [file Image_2.TIF]
